# Endoscopic blind limb reduction with septotomy: a novel endoscopic approach to candy cane syndrome after Roux-en-Y gastric bypass

**DOI:** 10.1016/j.vgie.2023.07.001

**Published:** 2023-08-30

**Authors:** Kambiz Kadkhodayan, Artur Viana, Sanmeet Singh, Dennis Smith, Shayan Irani, Dennis Yang, Mustafa Arain, Muhammad K. Hasan

**Affiliations:** 1 Center for Interventional Endoscopy, AdventHealth, Orlando, Florida; 2 AdventHealth, Seattle, Florida; 3 Virginia Mason Franciscan Health, Orlando, Washington; 4 Center for Interventional Endoscopy, AdventHealth, Florida

## Abstract

Video 1 The endoscopic blind limb reduction with septotomy procedure.

The endoscopic blind limb reduction with septotomy procedure.

## Case Presentation

We present here the case of a 51-year-old woman with a history of Roux-en-Y gastric bypass followed by surgical revision because of a nonhealing anastomotic ulcer. Her postoperative course was complicated by poor wound healing and multiple ventral hernias. She was referred to our practice with the diagnosis of candy cane syndrome, for which she was on parenteral nutrition for a year. After a multidisciplinary discussion, we decided to proceed with endoscopic treatment.

## Procedure Details

**Figure 1:**
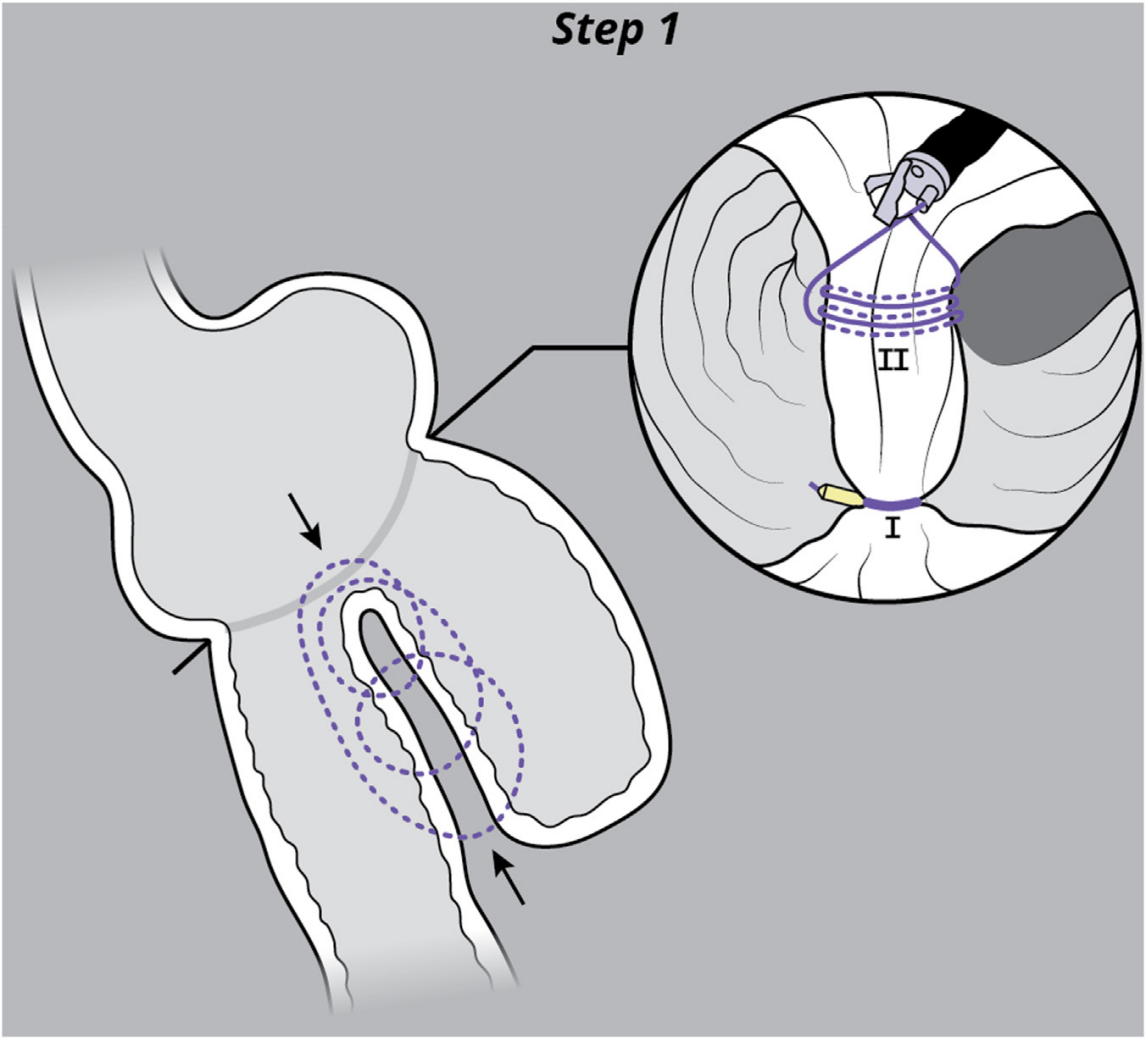
Illustration depicting step 1 of the endoscopic blind limb reduction with septostomy procedure; a single suture is applied at each end of the interjejunal septum. Multiple passes or bites are taken with each suture at each end before cinching.

**Figure 2:**
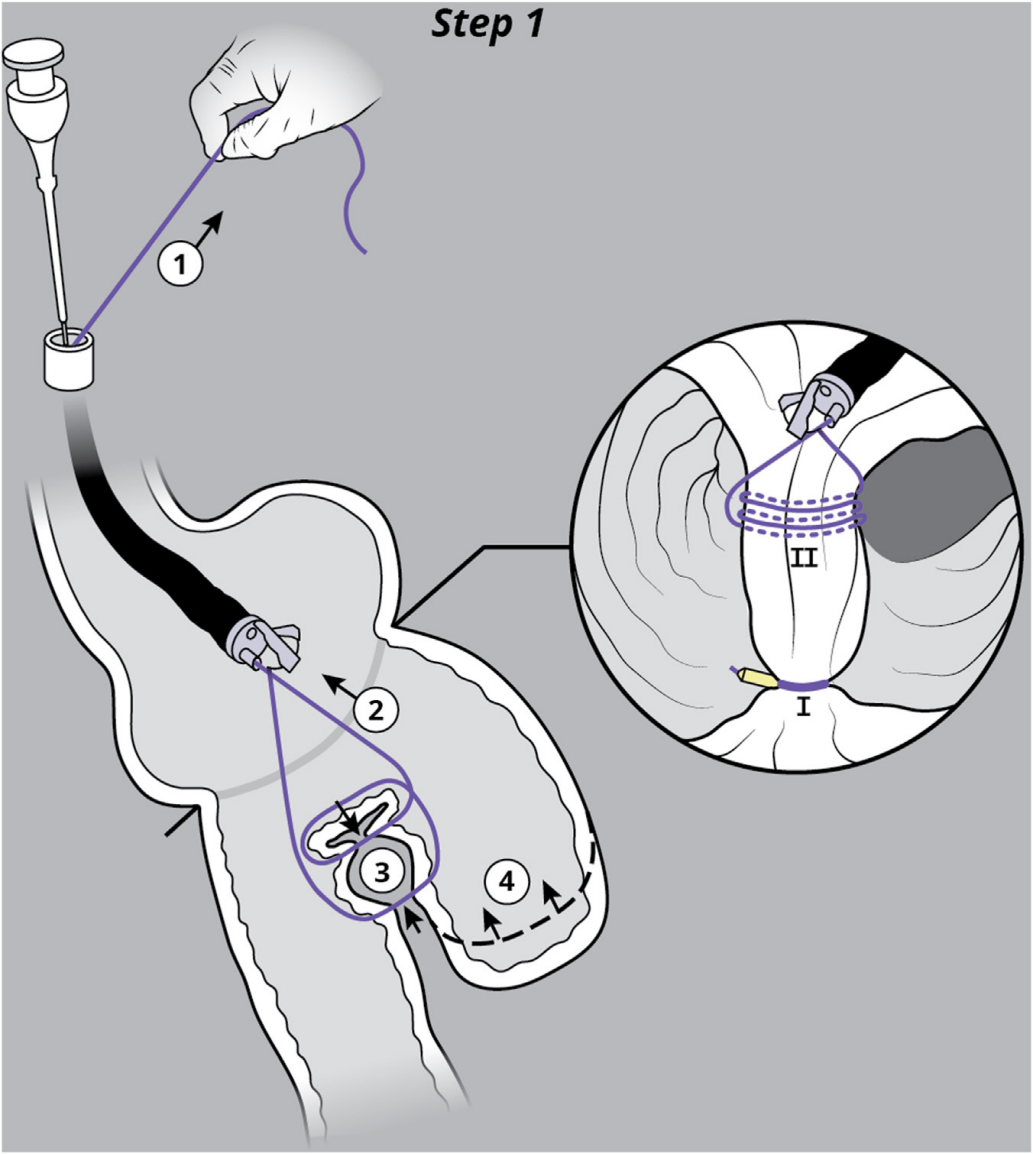
Illustration depicting the suturing technique used in step 1 of the endoscopic blind limb reduction with septostomy procedure. After each pass of the needle, constant external tension is applied on the suture (1). This results in tightening of the interjejunal septum between bites (2) and progressive shortening of the blind limb (3) until a desired blind limb length is achieved (4). Performing this on both sites of the interjejunal septum (I, II) results in blind limb reduction and creation of a septum that can be safely dissected using an electrosurgical knife.

**Figure 3:**
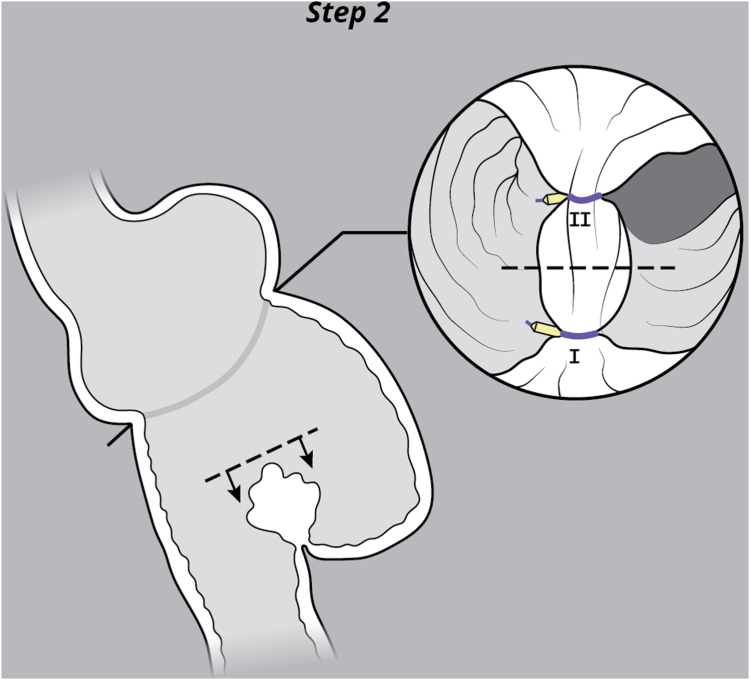
Illustration depicting step 2 of the endoscopic blind limb reduction with septostomy procedure; a scissor-type electrosurgical knife was used to dissect the interjejunal septum along a plane that is equidistant from and parallel to the sutures.

**Figure 4:**
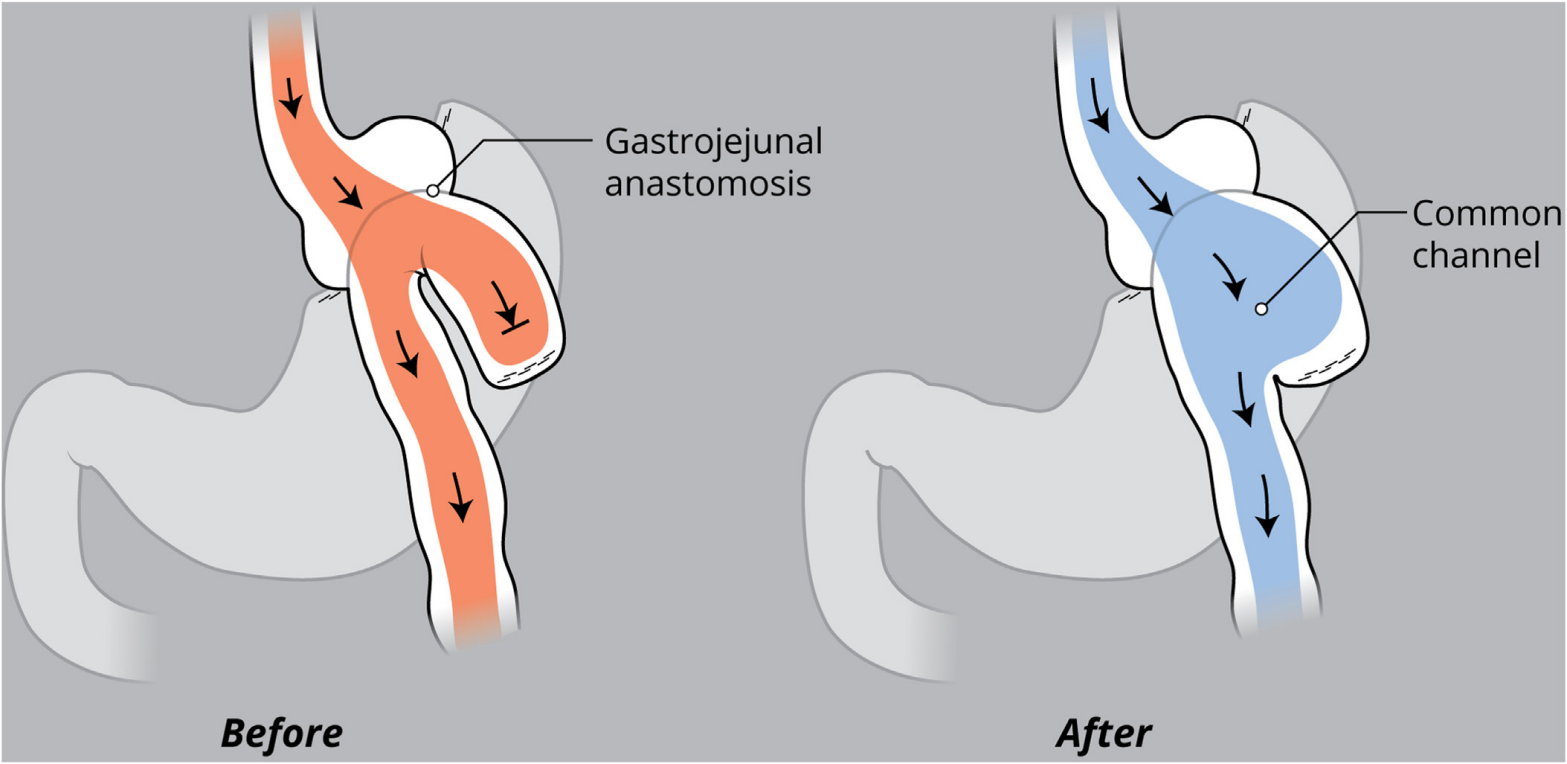
Illustration comparing candy cane–type anatomy (*before*) and the endoscopic blind limb reduction with septostomy procedure (*after*) that results in a common channel that connects the gastro-jejunal anastomosis to the afferent limb. *Arrows* depict direction of the enteric stream flow.

**Figure 5:**
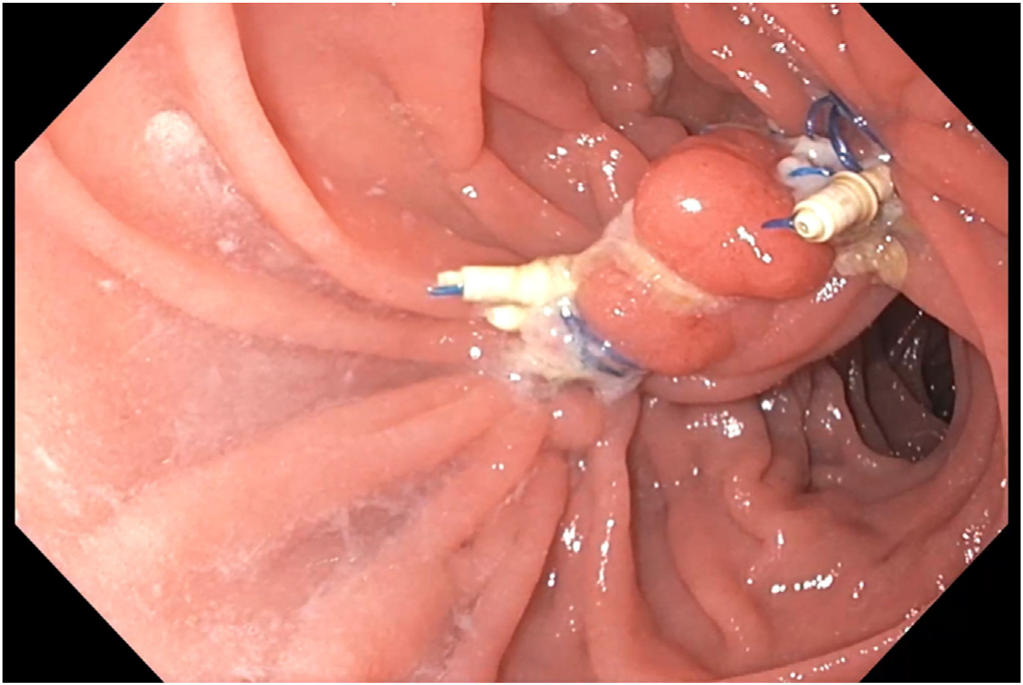
Endoscopic image taken 1 month after step 2 of the endoscopic blind limb reduction with septostomy procedure demonstrating near-complete absence of an interjejunal septum and blind efferent limb. A large common channel is visualized that leads directly into the afferent limb.

**Figure 6:**
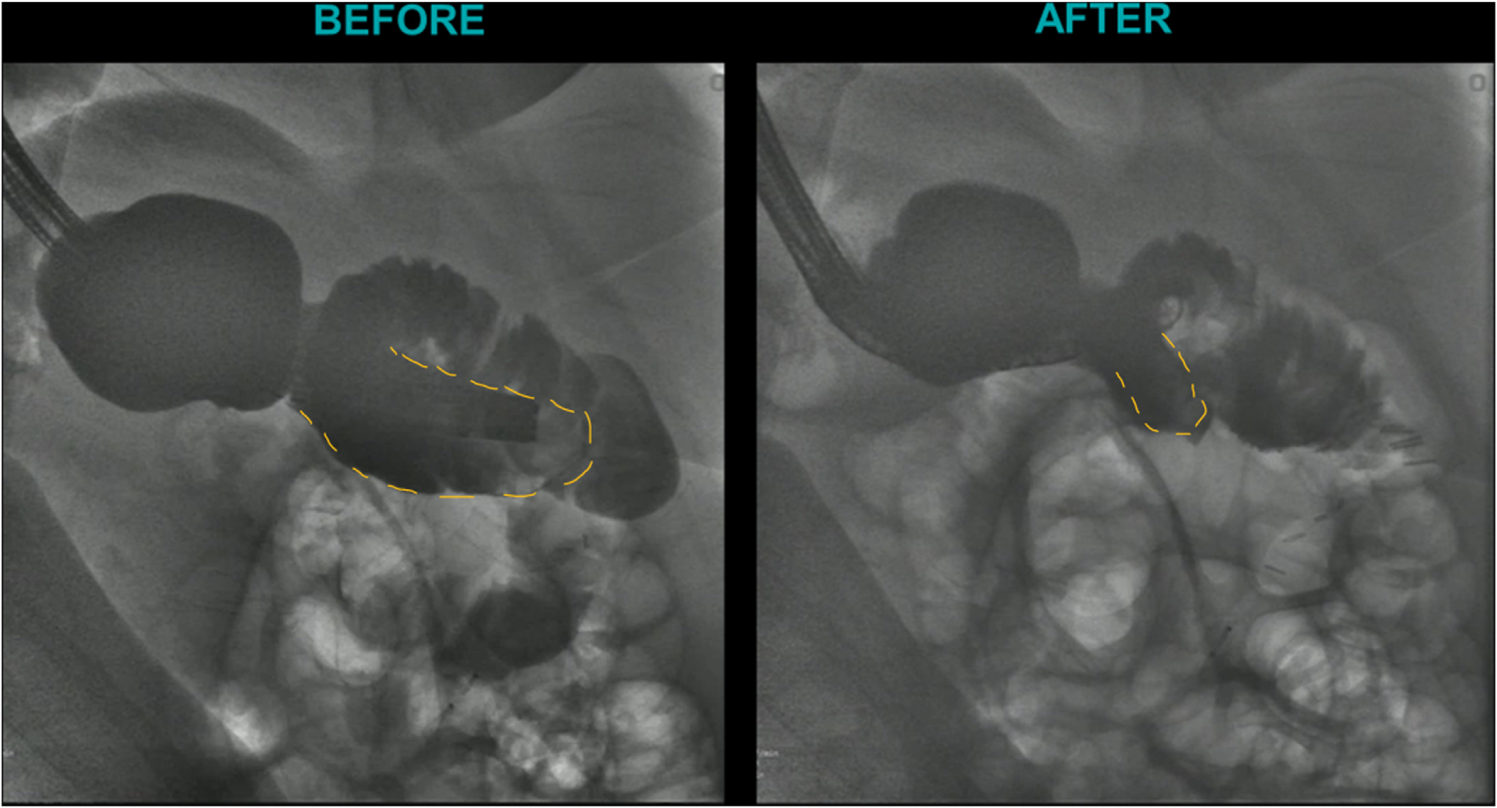
Fluoroscopic image after step 1 of the endoscopic blind limb reduction with septostomy procedure. Injection of water-soluble radio contrast demonstrates the typical candy cane–like appearance of a long blind afferent Roux limb (*yellow dotted line*) before the procedure. After step 1, we noted significant shortening and marsupialization of the limb (*yellow dotted line*).

**Figure 7:**
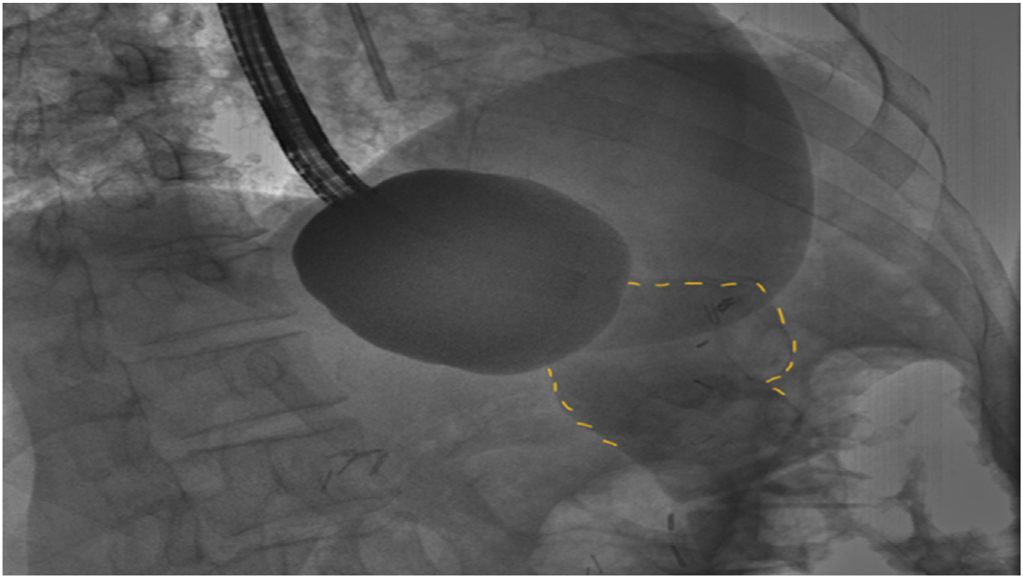
Fluoroscopic image after step 2 of the endoscopic blind limb reduction with septostomy procedure. Injection of water-soluble radio contrast demonstrates free flow of contrast through a common channel (*yellow dotted line*) into the afferent limb.

EUS was used to evaluate the interjejunal septum (IJS) and did not reveal intervening organ structures. There were, however, multiple medium-sized arterial blood vessels coercing through the IJS. We thus decided to proceed with endoscopic blind limb reduction with septotomy (ELR-S) using the following 2-step approach. In the first step, we used an endoscopic suturing device to place 1 suture at each end of the IJS. Multiple passes of the needle or bites were taken with each suture on either side of the septum (Fig. 1). Constant tension was applied on the external suture between each bite. This resulted in progressive shortening of the blind limb until a desired blind limb length was achieved (Fig. 2). The procedure was uneventful, and the patient was discharged the same day. The patient experienced significant improvement but continued to have residual postprandial symptoms. We then decided to proceed with step 2 of the procedure. On endoscopy, the IJS measured approximately 2 cm with minimal residual blind limb. EUS was performed and revealed no significant vessels within the IJS. After placing 2 additional sutures, the septum was dissected along a plane that was horizontal and equidistant from the 2 sutures. Dissection was carried out until the base of the septum was reached (Fig. 3). A hemostatic clip was applied at the apex of the septal dissection. Completion of the second step of the procedure resulted in creation of a common channel or pouch that extended from the gastro-jejunal anastomosis above to the afferent limb below (Figs. 4 and 5). Contrast was subsequently injected into the gastric pouch and revealed no leaks or perforations. When compared to the pre–ELR-S fluoroscopy, the blind limb was significantly reduced with no pooling and unrestricted passage of contrast into the afferent limb (Figs. 6 and 7).

## Discussion

Candy cane syndrome refers to patients who have undergone Roux-en-Y gastric bypass and develop significant and sometimes debilitating postprandial abdominal discomfort, which is often relieved by vomiting. This occurs because of an excessively long blind afferent Roux limb (BARL). Symptoms typically begin soon after a meal and dissipate after contents of the blind limb have either emptied into the afferent limb or been regurgitated.^1^ Revisional surgery, while highly efficacious, can be cost prohibitive and is frequently associated with a high risk of adverse events.^2^

Several innovative endoscopic techniques have been described that result in either diversion of enteric content from the blind into the afferent loop or shortening the BARL.^3^, ^4^, ^5^ We describe a novel 2-step procedure that simultaneously reduces the BARL length and diverts enteric content into the afferent limb. The procedure as described in this case report was uneventful (Video 1, available online at www.videogie.org), and the patient was discharged home the next day. On their 2-month follow-up, there was complete resolution of postprandial symptoms, total parenteral nutrition was discontinued, and the patient was able to tolerate an oral diet.

## Conclusion

We demonstrate feasibility of an endoscopic procedure that uses sutures to shorten the BARL and ligate the IJS at 2 ends. In our case, this led to significant improvement of candy cane symptoms. In addition, we demonstrate the feasibility of interjejunal septal dissection following placement of sutures. Further studies are needed to evaluate the procedure’s safety and clinical efficacy.

## Disclosure

Dr Yang is a consultant for Microtech, Medtronic, Olympus, FujiFilm, and Apollo Endosurgery. Dr Arain is a consultant for Cook, Merit, Boston Scientific, and Olympus. Dr Hasan is a consultant for Boston Scientific and Olympus. Dr Irani is a consultant for Boston Scientific, Gore, and CONMED. Dr Smith is a site proctor at Intuitive Surgical. All other authors disclosed no financial relationships.

## Supplementary data

Video 1 The endoscopic blind limb reduction with septotomy procedure.
